# A Cure for Sweating Feet

**Published:** 1889-08

**Authors:** R. W. St. Clair


### A CURE FOR SWEATING FEET.

BY R. W. ST. CLAIR, M. D.

I know of no disease more unpleasant, both to patients and those compelled to be with them. I have used, for several years, with the best results, a powder known as Streupulver. It is composed of three parts salicylic acid and eighty-seven parts silicate of magnesia. It is used in the German army for sweating feet. I have tried its efficiency in night sweats of consumptives, and am pleased with it. The beneficial effect is immediate and permanent. To prevent breathing the powder and the coughing it would cause, I place a cloth over the face while I rub the whole body with the powder. Once a day for three days, then every other day for a week, will be all that is required.

